# NiADA (Non-invasive Anemia Detection App), a Smartphone-Based Application With Artificial Intelligence to Measure Blood Hemoglobin in Real-Time: A Clinical Validation

**DOI:** 10.7759/cureus.65442

**Published:** 2024-07-26

**Authors:** Semanti Das, Farhad Ahamed, Aditi Das, Debjeet Das, Jhuma Nandi, Krishanu Banerjee

**Affiliations:** 1 Community Medicine, All India Institute of Medical Sciences, Kalyani, Kalyani, IND; 2 Community Medicine and Family Medicine, All India Institute of Medical Sciences, Kalyani, Kalyani, IND; 3 Pediatrics, All India Institute of Medical Sciences, Kalyani, Kalyani, IND; 4 Artificial Intelligence, Monere AI, Kolkata, IND; 5 Medical Imaging, Monere AI, New Jersey, USA; 6 Artificial Intelligence, Monere AI, Lehi, USA

**Keywords:** hemoglobin monitoring, smart phone app, image processing, ai, non-invasive, anemia

## Abstract

Background

Anemia is a severe public health problem in India affecting more than half of the population. To reduce its burden on the population, the Government of India under the Anemia Mukt Bharat program has adopted a monitoring strategy for the diagnosis and treatment of anemia. Point-of-care testing (POCT) devices play a pivotal role in testing hemoglobin at a community level where sophisticated laboratory instruments are not available. The majority of the currently available POCT devices are invasive in nature which have their own limitations. A non-invasive method of hemoglobin estimation will address many of the limitations of an invasive POCT instrument, which will further improve people’s acceptability for hemoglobin testing. The Non-Invasive Anemia Detection App (NiADA) (Monere AI Private Limited, Kolkata, West Bengal, India*)*, a non-invasive POCT application, uses artificial intelligence (AI) to predict the hemoglobin level from lower eyelid images. This real-time, point-of-care, low-cost solution uses a custom computer vision deep-learning algorithm to determine blood hemoglobin value.

Method

The study validates an AI-based smartphone application NiADA against laboratory hemoglobin estimation and a widely used point-of-care hemoglobin estimation instrument (HemoCue Hb 301; HemoCue AB, Angelholm, Skane County, Sweden). The study was conducted in a tertiary care hospital in Eastern India and recruited a total of 556 participants. These included 58 pediatric patients, 51 pregnant women, 214 adult females, and 224 adult males. Statistical analysis was performed using Python (Python Software Foundation, Wilmington, Delaware, United States). A p-value of < 0.05 was taken to be significant.

Result

The mean difference observed between NiADA and laboratory-estimated hemoglobin values came out to be -0.29 g/dL and -0.89 g/dL for adult females and males respectively, and 0.61 g/dL for pregnant women and -0.69g/dL for the pediatric population. The limits of agreement for NiADA were narrow at 2.77 to -2.18 g/dL for adult females, 3.76 to -1.96 g/dL for adult males, 1.89 to -3.29 g/dL for pregnant women, and 3.28 to -2.08 g/dL for the pediatric population. The sensitivity and specificity of the NiADA application against the laboratory estimation method were 75.8% and 53.8% for adult females, 70.0% and 48.3% for adult males, 23.8% and 90% for pregnant females, and 75% and 57% for the pediatric population.

Conclusion

As a non-invasive application, NiADA’s performance is satisfactory and comparable with minimally invasive tools like HemoCue Hb 301 and other POCT devices.

## Introduction

Anemia, the condition of inadequate red blood cells or hemoglobin [1], is a severe public health problem in India [2]. It has been found to cause multiple health problems leading to more than 50 million years of healthy lives lost due to disability [3]. The presence of anemia has also been associated with higher mortality and poor disease outcomes [4]. To address this huge burden, the Government of India, under the flagship program of Anemia Mukt Bharat, aimed to test and treat target age groups with the highest prevalence of anemia [5]. Prophylactic iron supplementation along with therapeutic dosing for anemic individuals is done under the program according to the prescribed guidelines. With advances in research regarding the various causes of anemia and the response of the body to iron, a widely acceptable method to monitor hemoglobin levels became necessary for testing and treatment of anemia at the community level with the help of healthcare workers namely, the Accredited Social Health Activists (ASHA), Auxiliary nurse midwife (ANM) and the Anganwadi workers (AWW). The current gold standard for laboratory estimation of hemoglobin is through hemoglobin cyanide (HiCN), the cyanmethemoglobin method, estimation in venous blood [6]. However, this method is expensive and complex and is not a feasible option in resource-limited settings [7]. The majority of the currently available methods, used for hemoglobin estimation including the gold standard and the point-of-care testing (POCT) devices, are invasive. 

The huge burden of anemia in India and the recent shift in strategy to test and treat requiring repeated blood collection and/or finger-pricks is very arduous, especially at the community level. The current POCT methods are limited by improper handling and maintenance by non-trained clinical staff, poor quality controls, lack of cost-effectiveness, insufficient documentation, and low comparability to laboratory methods [8]. An acceptable solution to these problems would be a non-invasive method that will make the process painless, real-time, smooth, and more accessible. Being non-invasive, such a method will also reduce the risk of infection, requirement of external equipment, chances of exposure to healthcare professionals, and biomedical waste generation [9,10]. These reasons have led to a growing interest in using non-invasive, POCT solutions for hemoglobin estimation in recent years.

A smartphone-based artificial intelligence (AI) application that determines real-time hemoglobin from the lower palpebral conjunctival images would not require any additional devices and would provide a low-cost alternative for the health system and a more acceptable and accessible method for the beneficiaries. The current study shall validate the non-invasive method of hemoglobin estimation through such an AI application against the gold standard venous blood hemoglobin estimation and a widely used POCT method. 

## Materials and methods

This was a validation study conducted among registered patients attending the outpatient department of a tertiary care hospital, All India Institute of Medical Sciences (AIIMS), Kalyani, West Bengal, India, from February 2024 to April 2024. The study was approved by the Institutional Ethics Committee, AIIMS, Kalyani (approval number: IEC/AIIMS/Kalyani/certificate/2024/04). Written informed consent was obtained from all the participants. Participant information was kept confidential and they could withdraw their consent at any point in the study without any loss of benefits being availed by them at the institute. The study complied with the 1964 Helsinki Declaration and its later amendments or comparable ethical standards.

Inclusion and exclusion criteria

The study included adult females, adult males, pregnant females, and the pediatric population. Severely sick or debilitated patients, patients with trauma to the eye, scarring over the eye or any other pathological or physiological condition of the eye that prevented photography of the lower palpebral conjunctiva, and all patients with hemoglobinopathies were excluded from the study.

Study procedure 

Registered participants who were eligible for the study were approached. Participants who consented to the study were interviewed regarding the sociodemographic details. Following the history taking, venous blood collection and capillary blood testing were done. After the venous blood collection, the microcuvettes of HemoCue Hb 301 (HemoCue AB, Angelholm, Skane County, Sweden) were filled from the second drop of capillary blood (from the middle or ring finger) for hemoglobin estimation. The manufacturer’s guidelines on the use of microcuvettes were followed. 

**Figure 1:**
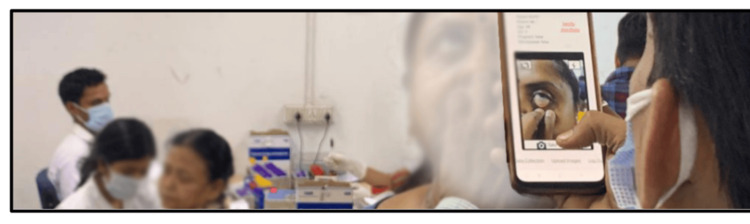
Lower eyelid image data collection using smartphone application, NiADA* *Monere AI Private Limited, Kolkata, West Bengal, India NiADA: Non-invasive Anemia Detection App

Finally, the patients were sat in a well-lit area. Trained personnel captured the bilateral lower palpebral conjunctival images on the smartphone (Figure 1). The smartphone needed to have the NiADA (Non-invasive Anemia Detection App) application (Monere AI Private Limited, Kolkata, West Bengal, India) installed and a good internet connection.

NiADA

NiADA is a smartphone application that predicts the blood hemoglobin level from the image of the lower palpebral conjunctiva. The application uses the built-in camera of the smartphone and hence no other extra device is required. The core technology behind NiADA is AI image processing. The custom machine learning algorithm has been trained by more than 30,000 sample lower eyelid images and corresponding hemoglobin levels. Along with the eyelid image, the application also collects the sex, age, and pregnancy status of the patient as input. 

**Figure 2:**
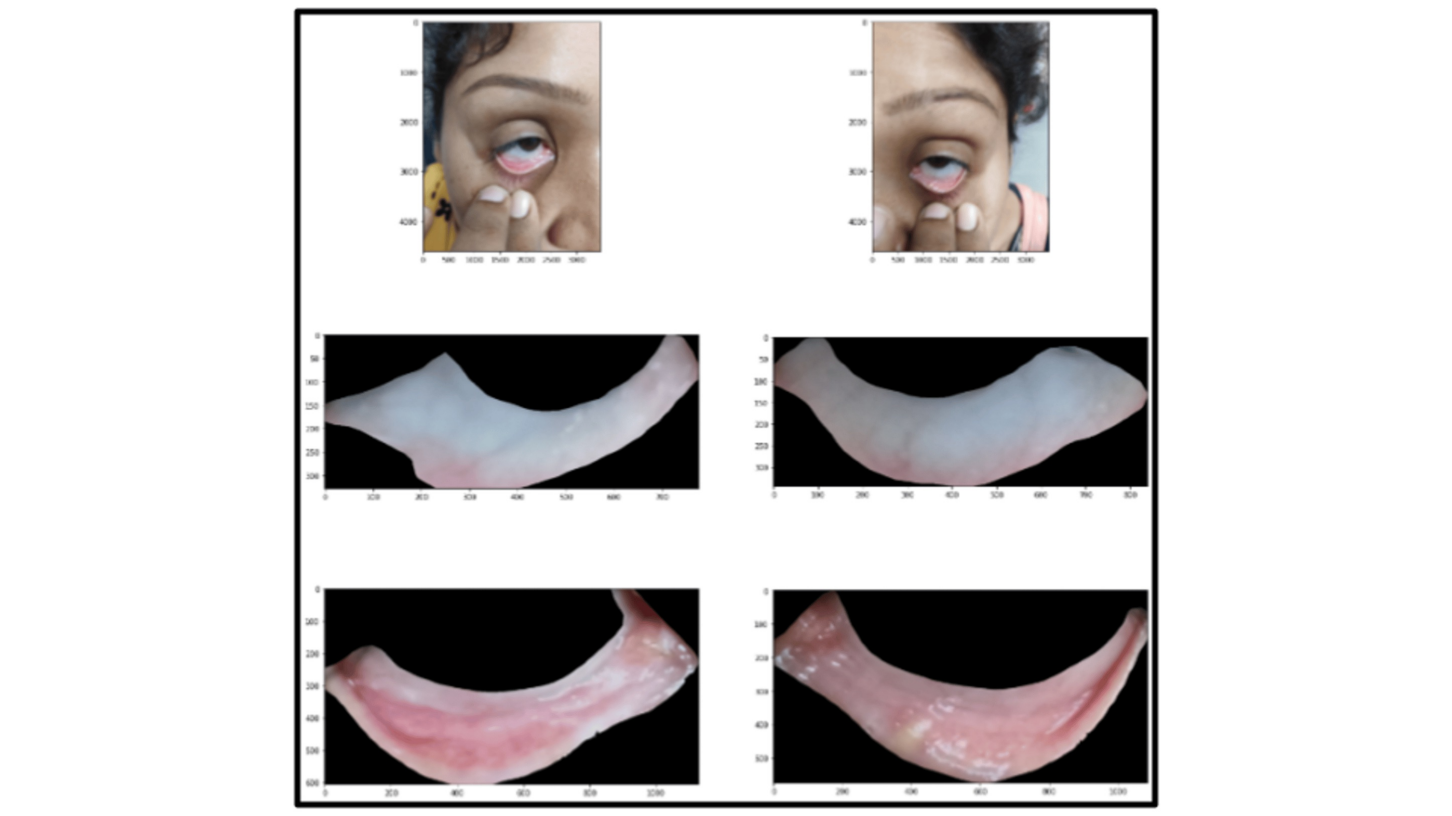
Eyelid and sclera segmentation from raw image Top row: Raw image taken; Middle row: Sclera; Bottom row: Eyelid

*Imaging and Image Processing* 

**Figure 3:**
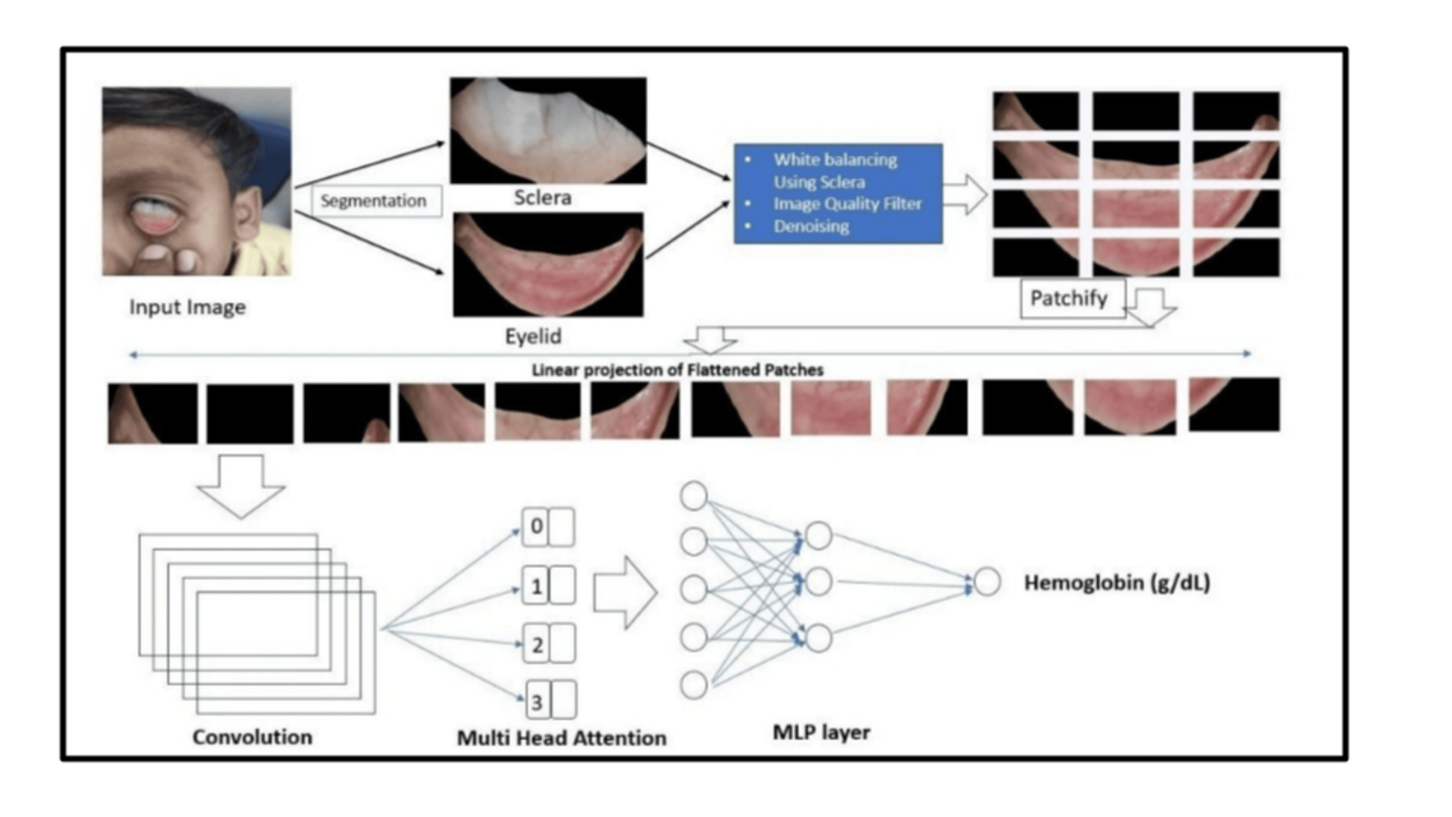
NiADA* workflow *Monere AI Private Limited, Kolkata, West Bengal, India NiADA: Non-invasive Anemia Detection App

Smartphone images were taken around six inches from a patient’s face. Images of both eyes were taken. A pre-developed segmentation model is used to segregate the portion of the image of the sclera and the lower palpebral conjunctiva from the images captured. To protect the privacy and data security of the patients, no other part of the eye image is used or stored in the application except the sclera and the lower eyelid. After the segmentation of the eyelid and sclera, the extracted region of interest is processed with several pre-processing steps before using as input for the hemoglobin estimation model. Pre-processing steps filter for very high illuminated, very low illuminated, not focused, or any other kind of low-quality images. Filtered images are then run through a regression model. This is a custom model, a combination of convolutional neural network (CNN) and vision transformer (VIT) used to find a contrast between different colors of the anterior and posterior part of the eyelid. The model results are confirmed by ten-fold cross-validation. The end-to-end flow of the NiADA model architecture is shown in Figure 3. 

Before delivering the result, the application compares the results obtained from the images of the two eyes. If the estimated hemoglobin values between the two eyes vary by more than 2.5 g/ dL then the application returns a message for recapturing the eyelid images. NiADA can store the historical values of the same patient.

Operational definitions 

The cutoffs for anemia diagnosis in the various age groups were according to the national program guidelines of Anemia Mukt Bharat [5]. For adult women, a hemoglobin of less than 12 g/dL was taken as the cut-off for the diagnosis of anemia. Adult males with hemoglobin levels of less than 13 g/dL were taken to be anemic. For pregnant women, hemoglobin less than 11.0 g/dL was taken as the cut-off for diagnosing anemia. In the pediatric population, for children less than five years a cut-off of 11 g/dL was considered for classification into anemia status, for children aged five to nine years it was 11.5 g/dL, and for adolescents, it was taken as 12 g/dL. 

Quality control 

Internal quality control (IQC) was run daily for the digital hemoglobinometer and the autoanalyzer (Sysmex Corporation, Kobe, Hyogo, Japan). All three levels of IQC samples (normal, low, and high) were checked and plotted in the Levy Jennings (LJ) plot. All values were found to be within the two-standard deviation range in LJ plot. The venous hemoglobin results were obtained from the National Accreditation Board for Testing and Calibration Laboratories (NABL) accredited laboratories. The hemoglobin status of the participants was shared with the principal investigator. Patients identified to be anemic by the auto analyzer were referred for treatment of anemia under routine care. 

Statistical analysis

Data was entered into the Epicollect5 mobile application (Centre for Genomic Pathogen Surveillance, Oxford, England) and analyzed using Python Language Reference, version 3.10 (Python Software Foundation, Wilmington, Delaware, United States). Continuous variables were reported through Mean (SD) and categorical variables through proportions. The Mean±SD of hemoglobin estimated in the NiADA application, HemoCue Hb 301, and Sysmex auto analyzer were compared. Paired analysis was done between hemoglobin values of NiADA versus Sysmex auto-analyzer and NiADA versus Sysmex auto-analyzer. The mean difference±SD between the NiADA and autoanalyzer and the limits of agreement were calculated based on the Bland Atman plot. Lin’s concordance coefficient was calculated with a 95% confidence interval (CI). Cohen’s Kappa coefficient was calculated to assess the agreement of the smartphone application for grades of anemia compared to the Sysmex auto analyzer. Sensitivity, specificity, positive and negative predictive values were calculated to assess the validity of the equipment. A p-value of < 0.05 was considered as the cut-off for ascertaining statistical significance. 

## Results

Study population statistics

A total of 607 samples were collected of which 51 proved to be unusable due to poor picture quality leading to a loss of 8.1% of the total available samples. A total of 556 participants were included in the final analysis of which 301 (53.9%) were females. The age of the population varied from four years to 80 years. A total of 214 (38.12%) adult females, 228 adult males (40.3%), 51 (9.2%) pregnant females, and 58 (10%) pediatric patients were included in the study. The number of pregnant females and pediatric patients included was 20% of the intended sample size due to the restricted footfall in the study period and refusal to consent on the part of the pediatric patients. The mean age of the adult females was 43.8±11.7 years and that of the males was 49.3±14.7 years.

Comparison between NiADA, HemoCue, and the laboratory analyzer

The mean hemoglobin for adult females through the NiADA application was 11.71±0.93 g/dL and it was significantly close (p < 0.05) to the mean hemoglobin values obtained from HemoCue estimation (11.32±1.56 g/dL) and laboratory estimation (11.98±1.04 g/dL); the mean difference in hemoglobin levels compared to HemoCue was 0.4 g/dL and for laboratory values was -0.27 g/dL. For adult males, the mean hemoglobin predicted by NiADA (12.66±0.83 g/dL) did not differ significantly from those predicted by HemoCue 301 (12.82±2.02 g/dL) but varied significantly (p < 0.05) from that estimated from the laboratory (13.56±1.72. g/dL). The mean difference in hemoglobin in males between HemoCue and laboratory values was -0.16 g/dL and -0.9 g/dL, respectively. In the case of pregnant females, the mean hemoglobin estimated through NiADA was 11.83±0.60g/dL which was higher than that estimated by HemoCue 301 (10.83±1.21 g/dL) and closer to the laboratory estimates (11.22±1.39g/dL) but the difference was statistically significant (p<0.05). The mean difference in hemoglobin predicted by NiADA was 1.08 g/dL and 0.70 g/dL for HemoCue and laboratory estimation, respectively. For the pediatric population, the mean hemoglobin predicted through NiADA was 11.81±0.69 g/dL and the value differed significantly (p< 0.05) from the laboratory estimated values (12.52±1.64 g/dL). There was no statistically significant difference from the values estimated through HemoCue (12.01±1.62 g/dL) in the pediatric population (Table 1).

**Table 1:** Mean±SD of hemoglobin determined by the NiADA, HemoCue Hb 301, and laboratory estimation among the study groups a = p < 0.05, b = p ≥ 0.05 NiADA: Non-invasive Anemia Detection App

	Adult females (n = 214)	Adult males (n =224)	Pregnant females (n =51)	Pediatric population (n= 58)
	Mean±SD values of hemoglobin	Mean±SD values of hemoglobin	Mean±SD values of hemoglobin	Mean±SD values of hemoglobin
NiADA	11.73 ± 0.94	12.66 ± 0.83	11.83 ± 0.6	11.81±0.69
HaemoCue 301	11.32 ± 1.57^a^	12.82 ± 2.02^a^	10.83 ± 1.21^b ^	12.01 ± 1.62^a^
Laboratory Analysis	11.98 ± 1.42^a ^	13.56 ± 1.72^b ^	11.22 ± 1.39^a^	12.52 ± 1.62^a ^

**Figure 4:**
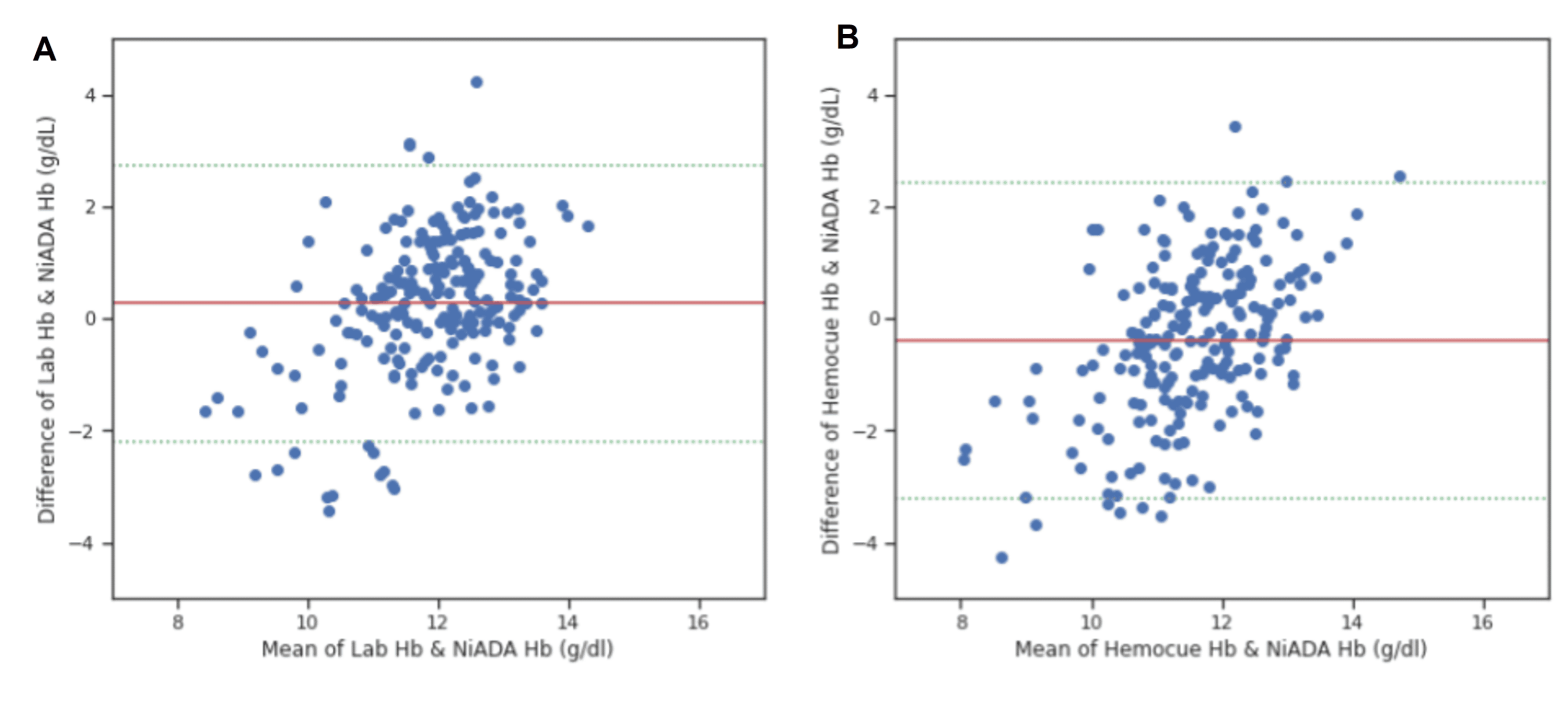
Bland-Altman plot for adult females. (A) Laboratory estimation vs. NiADA; (B) HemoCue vs. NiADA. NiADA: Non-invasive Anemia Detection App

**Figure 5:**
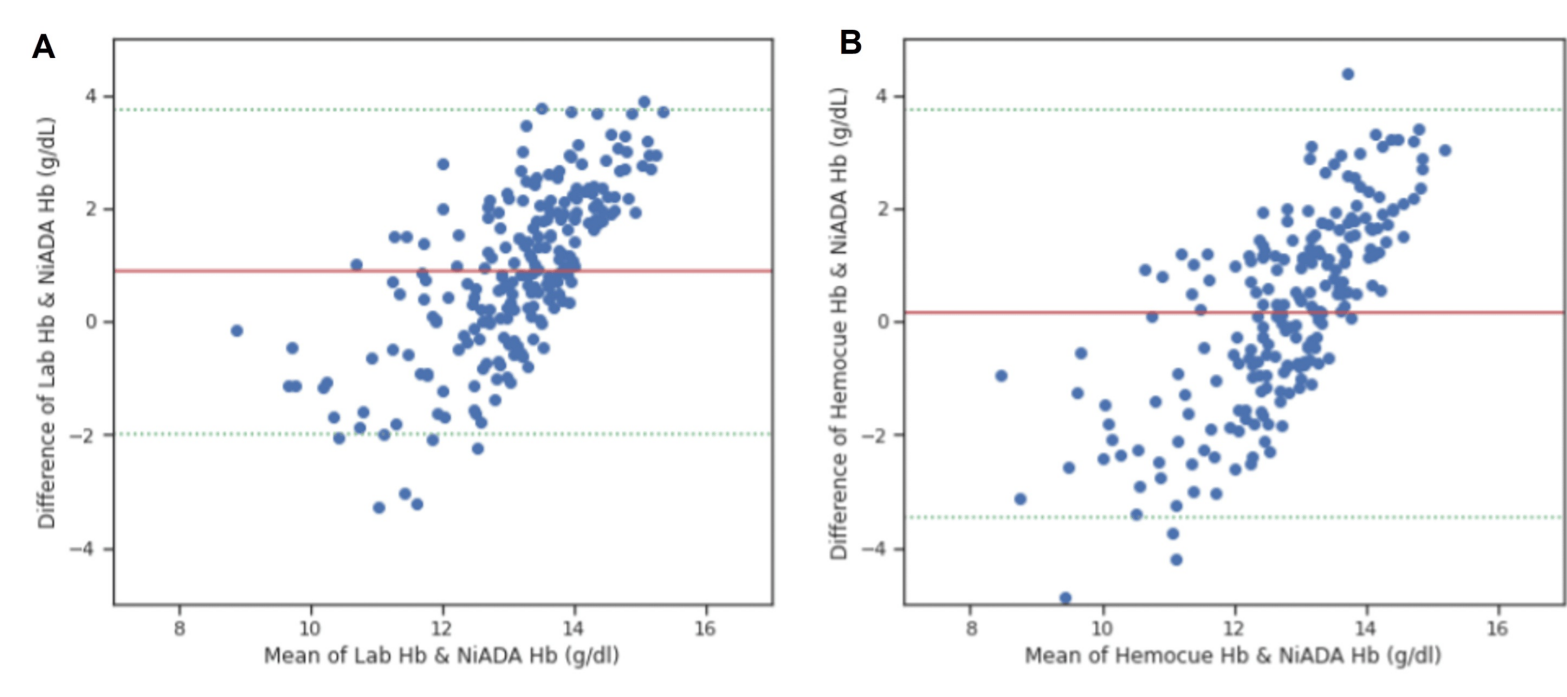
Bland-Altman plot for adult males. (A) Laboratory estimation vs. NiADA; (B) Hemocue vs. NiADA NiADA: Non-invasive Anemia Detection App

**Figure 6:**
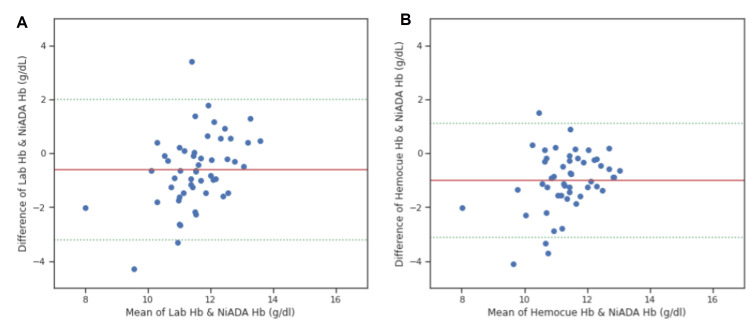
Bland-Altman plot for pregnant woman. (A) Laboratory estimation vs. NiADA; (B) Hemocue vs. NiADA NiADA: Non-invasive Anemia Detection App

**Figure 7:**
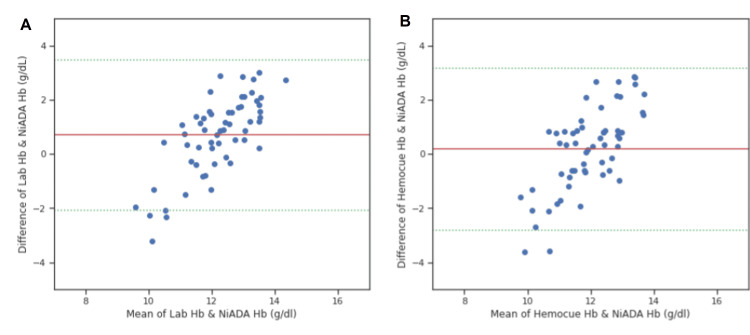
Bland-Altman plot for pediatric population. (A) Laboratory estimation vs. NiADA; (B) Hemocue vs. NiADA NiADA: Non-invasive Anemia Detection App

A Bland-Altman plot was constructed comparing the hemoglobin values estimated by NiADA against a digital haemoglobinometer (HemoCue 301) and Sysmex autoanalyzer and of HemoCue against laboratory estimation for adult females, adult males, pregnant females, and pediatric patients (Figure 4-7). NiADA had less bias (-0.29) but wider limits of agreement (2.77 to -2.18) when compared to HemoCue (Bias: -0.66 with limits of agreement 2.68 to -1.36) in the case of adult females. In the case of adult males, NiADA showed greater bias (-0.90) and wider limits of agreement (-3.76 to 1.96) in comparison to HemoCue (Bias: -0.73 with limits of agreement 3.25 to -1.79). For pregnant females, NiADA showed less bias (0.61) but wider limits of agreement (1.89 to -3.29) for laboratory analysis compared to HemoCue analysis (Bias: 1.08, Limits of agreement: 1.04 to -3.2). In the pediatric population, NiADA showed greater bias (-0.69) and narrower limits of agreement (3.28 to -2.08) against laboratory estimated values compared to HemoCue301 values (Bias: -0.32, Limits of agreement: 3.14 to -2.5). 

Correlation coefficient comparison

**Figure 8:**
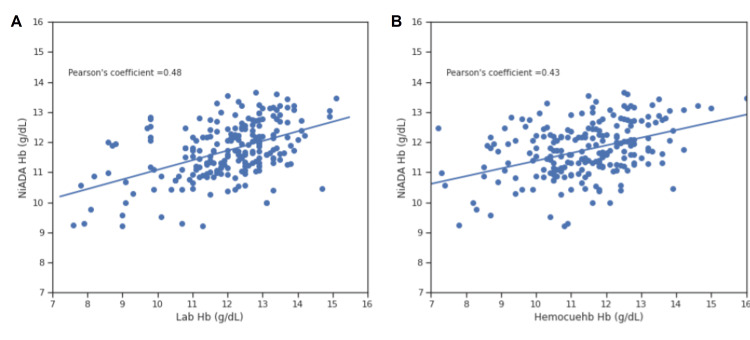
Correlation plot for NiADA compared to (A) Laboratory estimation and (B) HemoCue in adult females NiADA: Non-invasive Anemia Detection App

**Figure 9:**
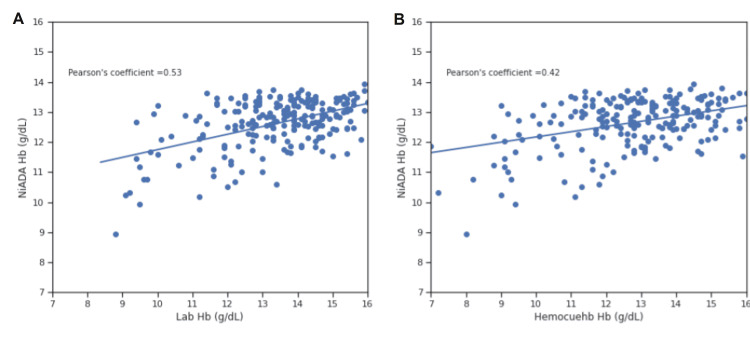
Correlation plot for NiADA compared to (A) Laboratory estimation and (B) HemoCue in adult males NiADA: Non-invasive Anemia Detection App

**Figure 10:**
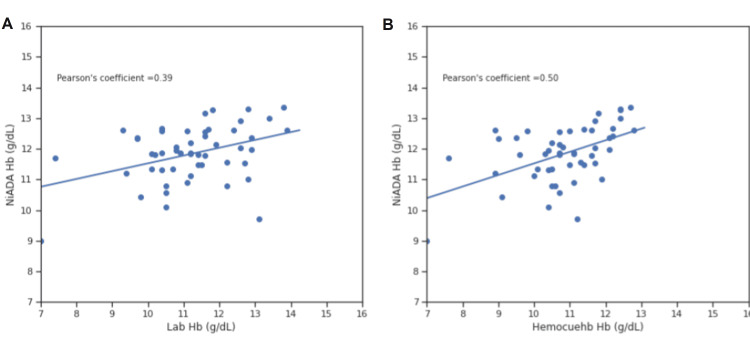
Correlation plot for NiADA compared to (A) laboratory estimation and (B) HemoCue in pregnant women NiADA: Non-invasive Anemia Detection App

**Figure 11:**
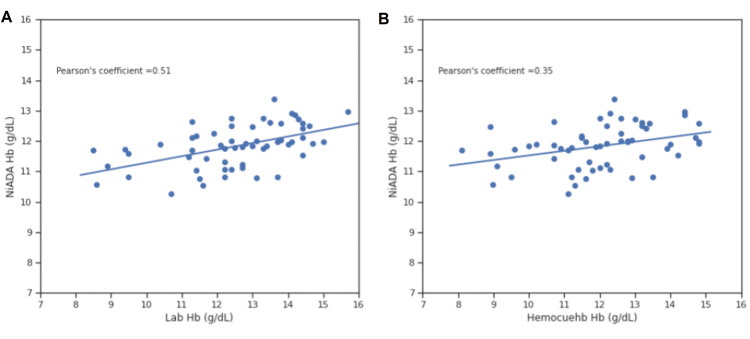
Correlation plot for NiADA compared to (A) Laboratory estimation and (B) HemoCue in pediatric population NiADA: Non-invasive Anemia Detection App

Figures 8-11 illustrate the Pearson correlation coefficient and Lin’s concordance coefficient for NiADA compared with HemoCue 301 and Sysmex analyzer (laboratory estimation). The Pearson correlation coefficient (ρ) for NiADA hemoglobin values showed a moderate correlation with laboratory values in the case of females (0.48), males (0.53), pregnant females (0.39), and pediatric patients (0.51). Lin’s concordance correlation coefficient (ρc) for NiADA when compared to laboratory values is 0.38 for females and 0.34 for males suggesting a fair correlation. For pregnant females and pediatric age group, Lin’s concordance correlation coefficient (ρc) came out to be 0.68 and 0.72, respectively, when compared with the laboratory hemoglobin values (Table 2).

**Table 2:** Bias (SD) of difference, limits of agreement, and concordance determined by NiADA, HemoCue Hb 301, and laboratory estimation among the study groups NiADA: Non-invasive Anemia Detection App

	Adult Females (n = 214)		Adult Males (n= 224)		Pregnant women (n =51)		Pediatric population (n =58)	
	NiADA and HemoCue	NiADA and Laboratory estimation	NiADA and HemoCue	NiADA and Laboratory estimation	NiADA and HemoCue	NiADA and Laboratory estimation	NiADA and HemoCue	NiADA and Laboratory estimation
Bias (SD) of difference	0.41 (1.47)	-0.29 (1.33)	-0.16 (1.83)	-0.90 (1.46)	1.08 (1.09)	0.61 (1.32)	-0.32 (1.44)	-0.69 (1.25)
Limits of agreement	2.47 to -3.29	2.77 to -2.18	3.75 to -3.43	3.76 to -1.96	1.04 to -3.2	1.89 to -3.29	3.14 to -2.5	3.28 to -1.62
Pearson correlation coefficient (ρ)	0.43	0.48	0.42	0.53	0.5	0.39	0.35	0.51
Lin’s concordance correlation coefficient (ρc)	0.34 (0.23- 0.43)	0.38 (0.28-0.48)	0.30 (0.21-0.37)	0.34 (0.26-0.41)	0.67 (0.53-0.77)	0.68 (0.53-0.79)	0.71 (0.55 -0.82)	0.72 (0.58 – 0.82)

Classification by cohorts

Although NiADA predicts hemoglobin value by regression model prediction, we can also use this prediction for classification purposes using threshold by cohort. On comparing the ability to detect anemia by NiADA application when compared to the laboratory estimation as the gold standard, the sensitivity of the application is 75.8%, specificity is 53.1%, positive predictive value (PPV) is 57.1%, and negative predictive value (NPV) is 73.3% in adult females. In adult males, the NiADA application shows a sensitivity of 70%, specificity of 48.3%, PPV of 43.1%, and NPV of 73.9% on comparison against laboratory estimation. In the case of pregnant women, the application shows sensitivity, specificity, PPV, and NPV of 23.8%, 90%, 62.5%, and 62.8%, respectively, while for the pediatric population, the test parameters came out to be 75%, 56.76%, 36%, and 87.5%, respectively (Table 3).

**Table 3:** Comparison of NiADA and HemoCue test parameters against laboratory estimation among the study groups NPV: negative predictive value; PPV: positive predictive value; NiADA: Non-invasive Anemia Detection App

Study group	Test parameters (%)	Hemoglobin assessment method	
		HemoCue Hb 301	NiADA
Adult Female (n = 214)	Sensitivity	95.6	75.8
	Specificity	55.8	53.1
	PPV	60.7	57.1
	NPV	94.7	73.3
Adult Male (n = 224)	Sensitivity	91.0	70.0
	Specificity	72.0	48.3
	PPV	62.8	43.1
	NPV	93.9	73.9
Pregnant women (n = 58)	Sensitivity	91.7	23.8
	Specificity	77.4	90.0
	PPV	75.9	62.5
	NPV	92.3	62.8
Pediatric patients (n = 51)	Sensitivity	75.0	75.0
	Specificity	75.7	56.8
	PPV	50.0	36.0
	NPV	90.3	87.5

The Cohen’s Kappa coefficient for NiADA in diagnosing anemia for adult females (< 12g/dL) was 0.227 against the gold standard laboratory estimation indicating a low level of agreement. In comparison with HemoCue, Cohen’s Kappa coefficient for NiADA was 0.186 indicating a low level of agreement. For pregnant females (< 11g/dL), Cohen’s Kappa Coefficient for NiADA and laboratory estimation was 0.121 and 0.125 for NiADA and HemoCue, indicating a low level of agreement. In pediatric patients, the Cohen’s Kappa coefficient was low at 0.229 and 0.233 for NiADA and HemoCue Hb 301 and NiADA and laboratory estimation, respectively. For adult males, the values came out to be 0.168 and 0.131 for agreement between NiADA and HemoCue Hb 301 and NiADA and laboratory values, respectively. 

## Discussion

The current study validated an AI-based imaging software application as a non-invasive method of hemoglobin estimation against the gold standard of venous sampling and laboratory estimation. The study also compared the results obtained from the smartphone application, NiADA, against the POCT instrument used in the Indian setting in the community-based health service provision, the HemoCue Hb 301. 

From the results, it is evident that for all cohort groups, NiADA shows significant agreement with laboratory estimation and the HemoCue Hb 301 with some exceptions. The low mean bias and limits of agreement make the NiADA application usable in community settings with reasonable confidence. Though the mean hemoglobin levels estimated by the imaging software NiADA were statistically significantly different from that estimated by laboratory estimation and the POCT device, HemoCue Hb 301, the difference was less than 1 g/dL. 

NiADA’s performance is comparable with other non-invasive hemoglobin measuring devices and even supersedes some of them. AnemoCheck(Sanguina, Inc., Georgia, United States) utilizes nail bed images to estimate hemoglobin [11]. AnemoCheck has reported true error likelihood of approval (LOA) ± 4.43 for adults and ± 3.54 for children. NiADA true error varies from -0.29 for adult females to -0.89 for adult males, which is much lower than ToucHb (Biosense Technologies Pvt. Ltd, Mumbai, India) [9]. NiADA's performance is also comparable with Radical 7 (Masimo Corp., Irvine, California, United States), which requires an additional expensive device [12]. The non-invasive Astrim Fit monitoring device (Sysmex Corporation) validated among school-going children showed a mean difference bias of 0.17 ± 1.95 g/dL and values of lower and upper limits of agreement as −3.65 and 3.99 g/dL [13]. The EzeCHeck (EzeRx Health Tech Pvt. Ltd., Bhubaneswar, Odisha, India) showed a moderate agreement between the non-invasive method and the gold standard method; 91.59% of the results lay within ±1.5 g/dL and 14.42% between ± 1 and ± 1.5 g/dL difference of lab hemoglobin estimation [14]. An imaging-based non-invasive hemoglobin estimation modality, sHEMO (Smartphone Spectroscopy for Blood Hemoglobin Level Monitoring), showed an accuracy of ±0.32 g/dL and sensitivity of 89% compared to the actual blood hemoglobin levels [15]. A diffused reflectance spectroscopy-based non-invasive and noncontact device (SAMIRA) showed Pearson’s correlation coefficient to be 0.987 for hemoglobin estimation against arterial blood-gas estimation [16]. Further operational research is required to understand the scalability and feasibility of these modalities in a heavy caseload-based setting, as in India. The photoplethysmography-based method of four-wavelength reflective oxygen sensor DCM08 for finger-end pulse wave signal detection showed a mean absolute error of 0.325 g/dl [17]. One of the major advantages of NiADA is that it does not need any additional device thus reducing logistic load and biomedical waste generation.

Hemoglobin estimation through eyelid color with different custom state-of-the-art) algorithms have also been explored. Many of them have achieved reasonable success. A study at Brown University with 202 patients achieved 72% accuracy [18]. Ghosal et al. listed some of the good approaches showing true error LOA less than ± 3 [15]. But most of them have tested on very low sample sizes. NiADA has been trained by more than 30,000 samples. Another major advantage of AI is its robustness along with scalability. 

In the present study, NiADA Pearson’s correlation coefficient compared to the laboratory estimation showed a good degree of association for pregnant women (0.4) and the pediatric population (0.53). In the Indian context, where there has been an increasing focus on the nutritional status of children and pregnant women, especially the anemia status of these target populations, a non-invasive portable device for hemoglobin measurement and information storage will prove to be very beneficial. Further operational research is required to understand the scalability and feasibility of the modalities in a heavy caseload-based setting, as in India. 

Monere is developing NiADA-V2 as an improvement to the current version through algorithm training, more samples, and further image processing, which shall address the issues faced in the preliminary version. The problems of low agreement and lesser correlation for certain hemoglobin ranges and populations shall be addressed in the upgraded NiADA version 2. 

There is a rising interest and ensuing research concerning non-invasive methods of hemoglobin estimation. The available evidence supports the non-invasive modalities as a substitute for laboratory and invasive hemoglobin estimation. Non-invasive methods, especially with the increasing role of AI in healthcare, shall prove to be a valuable boost to the testing and tracking of at-risk populations. India is steadily moving towards digitalization and the use of applications can help in the process. 

The current study was conducted in hospital settings by trained staff using ordinary smartphones. As hemoglobin is one of the fundamental biomarkers of human health, this real-time, non-invasive, low-cost solution will have a great impact on wellness and preventive healthcare. NiADA can be used as a screening device in point-of-care settings and can help in tracking patient's hemoglobin values. This shall boost the T3 approach adopted by the Anemia Mukt Bharat program as the application shall be usable at the community level by ASHA, ANM, and AWW. 

Strengths of the study include consecutive collection of the venous sample and capillary blood collection thus minimizing any within-person variations. Adequate quality control of the equipment was in place and monitored daily. The internal quality control of both the equipment (digital hemoglobinometer as well as the autoanalyzer) was done daily for all three levels of control and found to be within 2(SD) in the LJ plot. The approach of comparing capillary (digital hemoglobinometer) and venous (auto-analyzer) with the software application was adopted in the study to closely reflect the real-life use of these devices. 

Limitation of the study includes a lack of detected severe anemia cases by NiADA and lower accuracy in the extreme upper (>16 g/dL) and lower (< 8 g/dL) hemoglobin values. More samples (real and generated) and effective training of the software algorithm can help in addressing the problem. There were around 8% outliers that need to be addressed by further clinical examination or retesting. Improvement of image quality can reduce the percentage of outliers. It is also possible that blood hemoglobin color was not correctly reflected on the eyelid due to some other unknown medical conditions. The study was conducted in a healthcare facility setting by trained laboratory personnel. Thus, the results may change in the community setting where frontline workers would use this instrument. The application and hemoglobin values may be affected by the network connection and the phone camera quality. Appropriate mega-pixel format of the picture for accurate estimation needs to be explored. 

## Conclusions

The NiADA application by Monere is a viable option for community-based diagnosis and treatment of anemia because of its non-invasive nature. From the current study, it is evident that blood level hemoglobin can be estimated from the inner part of the lower eyelid images taken by ordinary smartphones. NiADA is not a replacement for laboratory hemoglobin testing. It is a screening and monitoring app. As hemoglobin is one of the essential biomarkers of human health, NiADA's real-time, non-invasive, low-cost solution will empower public administrators and other non-profit organizations around the world to screen and monitor anemia prevalence in an integrated way. NiADA can have a great impact on population health monitoring as it can be easily combined with nutritional planning and supplement distribution. It would provide a more ecologically sustainable and logistically feasible alternative to the current point-of-care devices used. In addition to that, NiADA can be a great tool at home to screen and monitor anemia for wellness and preventive purposes. As with AI applications, the dynamic model must be trained and updated regularly to provide accurate hemoglobin estimation. 
